# Mapping neonatal hearing screening services in Cape Town metro: A situational analysis

**DOI:** 10.4102/phcfm.v16i1.4386

**Published:** 2024-08-20

**Authors:** Petronella H. Louw, Tara Odendaal, Lebogang Ramma

**Affiliations:** 1 Department of Community Projects, Carel du Toit Trust, Cape Town, South Africa; 2 Department of Audiology, Carel du Toit Centre, Cape Town, South Africa; 3 Department of Communication Sciences and Disorders, Faculty of Health Sciences, University of Cape Town, Cape Town, South Africa

**Keywords:** hearing loss, neonatal hearing screening, low- and middle-income countries

## Abstract

**Background:**

Childhood hearing loss is a global health concern. Despite the proven benefits of neonatal hearing screening (NHS), it is not yet mandated in South Africa. The lack of awareness of hearing loss and absence of NHS leads to delayed diagnosis and adverse developmental outcomes for affected children.

**Aim:**

The study aimed to assess the availability of NHS services across primary healthcare (PHC) facilities in the City of Cape Town (CCT).

**Setting:**

Surveys were conducted with 26 PHC facilities in the CCT metropolitan areas that offer mother and child healthcare services.

**Methods:**

Surveys gathered data through online and telephone methods. The surveys aimed to assess the availability and nature of NHS services, care pathways and training of healthcare professionals regarding NHS.

**Results:**

None of the facilities used objective screening methods to screen hearing or have standardised care pathways for at-risk babies. Instead, they relied on parental concerns, with the use of the Road to Health book. None of the respondents reported having received hearing screening training, and the majority of participants (62%) lacked confidence in their knowledge of ear and hearing care.

**Conclusion:**

The absence of NHS services highlights the need for standardised protocols and increased awareness among healthcare workers and caregivers. Implementing NHS services could facilitate earlier diagnosis and intervention of hearing loss for infants in the Western Cape.

**Contribution:**

This study’s findings could guide efforts to improving access to NHS access at PHC level in Cape Town, ultimately providing early hearing screening services to infants.

## Introduction

Childhood hearing loss is considered the second most common disability worldwide,^1^ affecting at least 34 million children under the age of 15 years.^2^ The prevalence of childhood hearing loss is reported to be higher in low- and middle-income countries (LMICs) when compared to high-income countries, with sub-Saharan Africa (SSA) exhibiting one of the greatest prevalence rates of hearing loss.^3^ For instance, in the United States, the prevalence of childhood hearing loss is estimated to be 1 to 3 per 1000 live births.^1^ In contrast, countries like South Africa, where there are several of the known high-risk factors for congenital hearing loss such as poor maternal health, ototoxic drugs and premature birth,^2^ hearing loss is estimated to affect up to 3 to 6 per 1000 newborns.^2,4^

Unaddressed and late-identified hearing loss has long-term negative consequences on childhood development, which can result in speech and language delays, auditory processing difficulties, social communication difficulties, listening and attentional difficulties, poor quality of life and behavioural problems.^2,5^ Many of these children face barriers to access quality education, which can lead to low self-esteem and social isolation (socioemotional development) as well as poor academic outcomes and limited opportunities for future employment and participation in society. Hearing services are scarce in LMICs and not easily accessible in these settings. Public healthcare systems tend to prioritise more life-threatening diseases, thus overlooking hearing loss because of its non-life-threatening nature.^2^ Various factors contribute to limited accessibility, including insufficient awareness and understanding of hearing loss, societal prejudices, a lack of mandatory neonatal hearing screening (NHS) programmes, high costs associated with hearing screening equipment, and a shortage of trained professionals in hearing healthcare.^6,7,8,9,10^

While NHS programmes have clear benefits in improving outcomes for infants for congenital and early-onset sensory hearing losses, in LMICs the implementation is sparse because of severely limited access to hearing healthcare services in these settings.^2^ Timeous intervention is essential for enabling optimal development of speech and language skills, which form a cornerstone for the child’s cognitive, educational, social and emotional growth, as well as their acquisition of literacy skills.^5,6,7,8,9,10^ Internationally accepted standard for Early Hearing Detection and Intervention (EHDI) recommends detection of hearing loss by 1 month, diagnosis by 3 months and intervention by 6 months of age.^11^ To galvanise worldwide efforts for ear and hearing care, the World Health Organization (WHO) has pinpointed key indicators and established ambitious yet feasible goals, aiming for a 20% relative increase in the effective coverage of NHS services.^2^ The Health Professions Council of South Africa (HPCSA) adapted this standard to make it suitable for the South African undersourced context, recommending hearing detection by 6 weeks, diagnosis by 4 months and intervention no later than 8 months.^12^

Owing to resource constraints, targeted screening where babies having higher risk factors for hearing loss are screened is the recommended approach to NHS in some LMICs.^12^ In South Africa, despite the recognition of the importance of EHDI services by the HPCSA, the absence of legislation for NHS, coupled with a lack of awareness regarding its significance, often leads to significant delays in diagnosis with average ages of first diagnosis ranging from 23 to 42 months of age.^13,14^

In LMICs, hearing screening across the lifespan plays a critical first opportunity to identify children with a range of hearing loss – from congenital and early-onset sensory losses to late-onset, progressive or fluctuating types, including both permanent and transient conditions; these screenings address a crucial gap in healthcare provision for children in these settings.^7,15,16,17^ Significant challenges to accessibility remain in LMICs, including geographical obstacles and inadequate healthcare services. Thus, travel for follow-up assessments and interventions is burdensome towards the patient and their family.^6,18^ Accessibility is not just physical; it is a widespread problem encompassing the affordability of indirect costs such as transportation and time.^19^ Screen refusal and coverage are also influenced by caregiver understanding and perspectives towards NHS, both of which could be improved through support of the NHS by healthcare professionals involved in the newborn’s care.^13^ Even in instances when hearing services are nominally free, the related indirect costs can still be prohibitive, resulting in missed follow-up appointments and the lack of understanding of the importance of ongoing care.^13,20,21^ Delayed follow-up exacerbates the diagnosis and treatment of hearing loss, which may have a detrimental effect on childhood development and later academic progress.^22^

The healthcare system in South Africa consists of a state-run and taxpayer-funded public healthcare sector as well as a private sector serving individuals covered by private medical insurance or those who pay for care themselves. Approximately 16% – 45% of the population utilises private healthcare, while 55% – 84% rely on public healthcare facilities.^23,24^ Inadequate equipment, training and staff shortages strain the public healthcare sector, leading to over 90% of infants in South Africa lacking access to the NHS.^25^ Caregiver knowledge and attitudes towards the NHS also influence screening acceptance and coverage, highlighting the need for healthcare professionals’ support in promoting awareness and accessibility of the NHS for newborns.^2,26^

In LMICs like South Africa, healthcare is tiered into three levels: primary, such as point-of-entry clinics, secondary, which includes district and regional hospitals, and tertiary, which includes specialised services.^27^ Limited hearing screening performed at primary level facilities results in direct referrals for initial hearing screening to centralised tertiary level hospitals, contributing to increasing waiting periods in specialised care such as diagnostic hearing assessments and intervention services. This hampers timeous access to hearing healthcare services in resource-constrained communities in LMICs.^2,7,13^ This direct referral system can also mean that parents and caregivers must travel far distances for access to hearing healthcare, resulting in poor follow-up rates, delayed diagnosis and late access to hearing technology. Decentralisation of hearing healthcare services is crucial for sustainability. It involves shifting responsibility from central to local (primary) levels and requires addressing socioeconomic barriers.^28^ Without hearing healthcare available at primary level clinics, many communities lack access altogether, while tertiary level services become overloaded with screening tasks that could be handled at lower levels of care.^29^ Prioritising community-based care can alleviate burdens on tertiary facilities and improve accessibility.^30^ With a substantial portion of childhood hearing loss being preventable, integrating health promotion activities is vital.^2^ Awareness and education among caregivers regarding the NHS are crucial for its effective implementation. The extent to which caregivers understand the importance of the NHS directly influences their involvement and active participation. Public awareness initiatives at appropriate levels, for example, antenatal visits, particularly in LMICs, are essential for the success of NHS programmes in South Africa.^13,31^

Currently, in South Africa, the most common way to identify children with hearing loss often relies on caregivers raising concerns about their perceived delays in their child’s hearing, speech and language development.^32^ Relying on subjective identification leads to children with hearing loss being identified too late (after 4 years),^2,8,9^ because by that time the child has missed the critical time window to benefit from the most effective methods of intervention for communication development.^15,33^ Therefore, there is a need to raise awareness and advocate for improved access to the NHS in South Africa. Parental or caregiver concern about their child’s hearing and communication development should warrant an immediate referral for a hearing assessment. Parental concern is of greater predictive value than the typical informal screening done at primary healthcare (PHC) level.^34^ A previous study showed that parents were as much as 12 months ahead of physicians in identifying their child’s hearing loss.^34^ Another study found that although parents often underestimate hearing problems in their children, their sensitivity of detecting hearing loss reached as high as 20% for mild hearing loss and above 30% for moderate or more significant hearing loss.^35^ Thus, caregiver concerns about hearing should be prioritised, particularly in resource-constrained settings where access to objective hearing screening is limited.

To enhance the advocacy efforts to improve the availability and accessibility of NHS services in the City of Cape Town (CCT), it is important to ascertain the current status of these services at PHC facilities, which serves the vast majority of people residing in the CCT. Specific objectives of the study were to describe the nature of NHS services provided at PHC facilities, determine the current care pathway for babies who are identified with potential risk of hearing loss, and assess the awareness, knowledge and training of PHC providers working at PHC facilities with respect to hearing health in babies younger than 1 year old.

## Methods

### Study design

Two descriptive online surveys were used to collect data relevant to this study. The surveys were developed in Google Forms by the researchers using existing literature and questions that arose from practical experience in the field of this study.^8,14,15,18,26,31,36,37,38,39,40^ The surveys focused on assessing the availability of resources (e.g. staff and equipment) for hearing screening at the facility, referral protocols and pathways, participants’ awareness and knowledge of hearing and communication development, the risk factors associated with hearing loss, and the participants’ level of training received on hearing healthcare and hearing screening.

### Study setting

The study was conducted in the CCT metropolitan area, which is home to approximately 4 million people, with about 80% of the population utilising public health facilities.^23,41^ The CCT: City Health Department includes four areas, namely, Area North (Western and Northern subdistricts), Area South (Mitchells Plain and Southern subdistricts), Area Central (Klipfontein/Tygerberg subdistricts) and Area Eastern (Khayelitsha and Eastern subdistricts). A total of 55 of the CCT healthcare clinics, where mother and child healthcare services are offered, were identified as potential health facilities to participate in this survey.

### Study population and sampling strategy

The study population consisted of nursing staff (clinic managers, assistant managers, professional nurses) working in City Health PHC Facilities that provide mother and child healthcare services. The survey was completed by a single representative from each facility. Participants were sampled through purposive sampling, ensuring the inclusion of individuals who met specific criteria. Eligible participants were those actively engaged in healthcare delivery at a facility providing mother and child services, and specifically offering care to infants under the age of 1 year.

### Pilot study

Before commencing data collection, the newly developed surveys underwent a pilot study to ensure coherence and relevance to the study objectives. Feedback from six experienced healthcare professionals in the field of hearing screening, including audiologists and two hearing screeners, helped simplify and adjust survey questions. The pilot study served to achieve three objectives: to assess the clarity and comprehensibility of the survey items, to determine the time required for respondents to complete the survey and to verify the functionality of the online Google survey link. Based on the feedback received, the surveys underwent review and adaptation to refine them for use in the study.

### Data collection procedure

Following obtaining ethical approval and permission from relevant authorities, the contact details of 55 facilities that were identified and selected to participate in this study were obtained from the Western Cape Government official website. An email was addressed to the facility manager, inviting one person from each facility to take part in the study. The email outlined the purpose of the study and included a link to complete the online survey. The participants were required to provide consent and were given two weeks to complete the survey. They were also required to complete the survey directly online using Google Forms. Follow-up telephone calls (contact numbers were obtained from the CCT) were made to the facilities when the respondent rate was low after 2 weeks. Telephone conversations were conducted with one person from each facility, which included clinic managers, assistant managers and professional nurses. The surveys were conducted in English and participants provided written or verbal consent to validate their participation in the study. On confirmation of consent, the survey was carried out telephonically by the research assistant, which took between 10 and 15 min to complete. The research assistant completed the survey verbatim as per their responses during the telephone call. All survey information was captured manually in hard copy by the research assistant and was later recorded electronically for analysis.

Two different surveys were developed and aimed at two different subgroups:

Survey A: Facilities that see babies younger than 1 year old but do not offer hearing screening (with equipment) – see online supplemental material A.

The survey consisted of 45 items in total: 3 open-ended questions and 37 close-ended questions. Five additional items were provided for additional comments and other alternatives (to the options provided). Multiple choices were provided for completion of the close-ended questions and statements.

Survey B: Facilities that see babies younger than 1 year old and do offer hearing screening (with equipment) – see online supplemental material B

The survey consisted of 50 items in total: 2 open-ended questions and 42 close-ended questions. Eight additional items were provided for additional comments and other alternatives (to the options provided). Multiple choices were provided for completion of the close-ended questions and statements.

### Data management and analysis

Completed surveys from the participants were securely captured using a Google Form and subsequently imported to a Microsoft Excel spreadsheet for analysis. In addition, the answers from the telephonic surveys were transcribed and de-identified and collated onto the same Microsoft Excel spreadsheet. Data were analysed using descriptive statistics to obtain a concise overview of the gathered data and identify potential patterns within the participants’ responses. Proportions, expressed as percentages, were used to report the data, and graphical representations were utilised to visually present the findings.

#### Participants description

The survey link was sent to 55 CCT PHC clinics that provide mother and child healthcare services, and only 26 of the 55 participants (47%) agreed to participate in the study. Majority of the participants (18/26; 69%) were employed as professional nurses. Refer to Table 1 for a detailed description of the participants.

**TABLE 1:** Socio-demographic characteristics of participants (*N* = 26).

Variables	Percentage (%)	Number of participants (*n*)
**Primary language**		
Afrikaans	46	12
isiXhosa	27	7
English	19	5
Other	8	2
**Job category**		
Professional nurse	69	18
Management	27	7
Community healthcare worker	4	1
**Location of the facilities (and participants) inside the CCT**		
Northern area	15	4
Southern area	19	5
Central area	35	9
Eastern area	31	8

Note: Rounding was done on the totals and percentages.

CCT, City of Cape Town.

### Ethical considerations

An ethical clearance for this study was sought and obtained from the University of Cape Town Human Research Ethics Committee (HREC No. 229/2022) prior to the start of data collection. Permission and approval to conduct the study were also sought and obtained from the City Health Department (#9540). Following a clear explanation of the purpose of the study and use of data, all facility managers and participants gave informed verbal consent during the telephone interviews and informed written consent in the Google Forms to take part in the study. All responses were captured electronically and de-identified to maintain the confidentiality of data. The confidentiality of participants’ identities and personal information was strictly maintained throughout the study. Each participant was assigned an alphanumeric code, which was used in the Microsoft Excel spreadsheet and all related documentation to ensure privacy. All participants were informed that their identities would remain strictly confidential. Information disclosed during the telephonic survey and the results obtained were known only to the researcher and supervisor. Any identifying data were omitted from the Microsoft Excel spreadsheet to further ensure privacy. Only the interviewer and the researcher were aware of the participants’ identities. Although participants’ home languages were different, they all expressed good understanding of the English questions.

## Results

Some of the participants agreed to participate and completed the online Google Forms survey, and others preferred to complete the survey telephonically. Out of the 55 facilities that were contacted, 33% (18/55) of the participants (from different facilities) completed the survey telephonically and 14.5% (8/55; from different facilities) completed the survey online, with a total of 52.7% (29/55) being unreachable.

### Nature of neonatal hearing services currently offered in the public health sector (primary healthcare level) in the City of Cape Town (Figure 1)

Only a small number of participants (8%; 2/26) indicated that their facility offered NHS services at their facilities and thus completed Survey A. These two participants reported that they use the Road to Health booklet developmental screening tool and feedback from the parents as a method of obtaining information about the babies’ hearing development. One of these participants reported incorporating the use of a rattle test as hearing screening method which is not a standardised objective method of screening a baby’s hearing. However, neither of the two participants reported using objective hearing screening methods such as the otoacoustic emissions (OAEs) or automated auditory brainstem response (AABR) tests. Majority of participants (92%, 24/26) indicated that they do not have equipment for the NHS at their facilities and thus completed survey B. Based on the responses obtained from all the facilities, there was in fact no facility that offered objective hearing screening services with equipment. Hearing screening services primarily relied on verbal inquiries made to parents and caregivers concerning their child’s hearing and communication development with the use of the Road to Health booklet.

**FIGURE 1:**
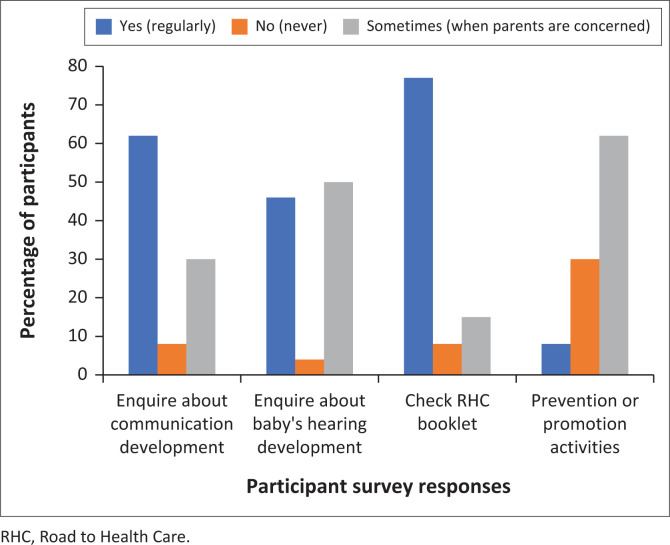
Screening methods for a baby’s hearing and communication development (*N* = 24).

Among the participants who reported that they did not have hearing screening equipment at their facility, less than half (46%; 11/24) reported that they do inquire about the baby’s hearing development at every clinic visit, and only one participant (4%; 1/24) reported that they do not ask the parents specifically about their baby’s hearing development while the other half (50%, 12/24) reported that such inquiries were conducted on an occasional basis or done only when the parent or caregiver expressed concern about the child’s hearing. In the study, 62.5% (15/24) of participants reported consulting parents or caregivers about communication concerns, while 29.2% (7/24) did so occasionally or upon parental expression of concern. Only 8% of participants (2/24) did not enquire about communication milestones. A third of participants (33%; 8/24) reported that they do not have any hearing-related prevention and promotion activities at their facilities. Sixteen of the participants (67%) do offer some prevention and promotion activities while two (8%) reported having regular sessions (raising awareness on hearing loss) at their facilities. Refer to Figure 1 for a summary of the screening methods used for a baby’s hearing and communication development at PHC facilities.

### Current care pathway for babies who are identified with potential risk for communication or hearing loss (Figure 2)

**FIGURE 2:**
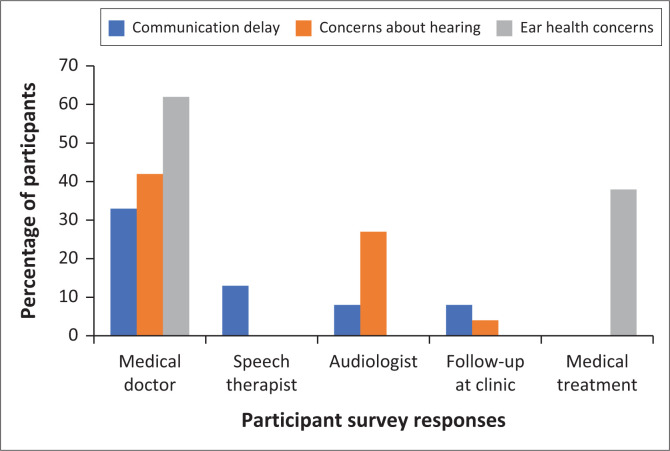
Referrals for a baby’s ear health, hearing and communication concerns.

All participants indicated that they do make referrals when the need arises. However, there was no standardised referral pathway for children who are identified with possible communication delays or hearing loss. A third of participants (33%, 8/24) reported that they referred a parent or caregiver to a medical doctor when there are concerns about the child’s babbling (communication development). A small number of participants (12.5%; 3/24) reported directing referrals to a speech therapist, with an additional two participants (8%; 2/24) indicating their preference for referring exclusively for a hearing test. Interestingly, two participants (8; 2/24) reported that they would consider the need for a further assessment once the infant reaches 1 year of age. Figure 2 provides a summary of the referrals and management offered at the PHC facilities for babies who are suspected of hearing loss, communication delays and ear health concerns.

### Awareness, knowledge and training of primary healthcare providers regarding hearing health in babies younger than 1 years old (Figure 3)

Majority of participants (73%; 19/26) in this study indicated that they had not received any specific training on children’s ear and hearing care. Only 27% (7/26) reported having received such training, with most of them (71%; 5 out of 7) being students at the time. More than a third of participants (38%; 10/26) expressed confidence in their knowledge of ear and hearing care, while the majority of participants (62%; 16/26) indicated that they were not confident in their knowledge, albeit some of them (31%; 8/26) feeling capable of providing assistance to babies. All of the participants affirmed the importance of early detection of hearing loss, stating that screening can feasibly occur before infants reach 6 weeks of age.

**FIGURE 3:**
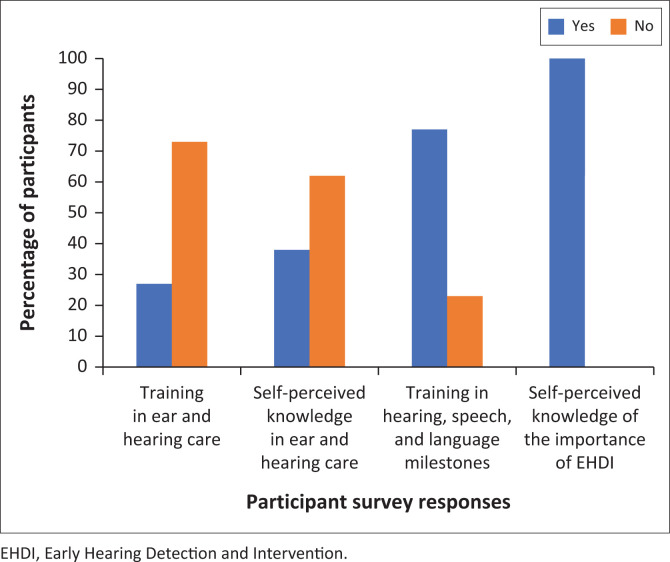
Self-perceived knowledge and training in hearing healthcare.

Of those who indicated they do not offer hearing screening with equipment at their facility, most of the participants (83%; 20/24) indicated that they had not received any training in hearing and communication milestones in babies and children, and the remaining 17% (4/24) had received the relevant training. Figure 3 provides a summary of the participants’ self-perceived knowledge and training of ear and hearing care, and related developmental milestones in babies and children.

On average, participants accurately identified 89.5% of risk factors for congenital hearing loss and correctly addressed primary ear health concerns, as indicated by their scores on the close-ended, multiple-choice questions. This high score indicates a strong self-perceived awareness, knowledge and training in the field of primary ear and hearing care understanding of these areas among the participants. Furthermore, all participants indicated a keen interest in expanding their knowledge and training on primary ear and hearing care.

## Discussion

This study aimed to investigate the availability of NHS services across PHC facilities in the CCT. Study findings emphasise the importance of the NHS and the need for standardised protocols and increased awareness among healthcare workers and parents/caregivers.^31,36^ The surveys conducted in this study indicate that none of the PHC facilities perform routine hearing screenings (with equipment) on babies. Furthermore, there is a lack of standardised referral pathways for babies with suspected hearing loss, and the majority of participants had not received formal training in primary ear and hearing healthcare. A considerable portion of births occur outside of public healthcare hospitals; thus, it has been suggested that immunisation clinics serve as a suitable venue for community-based infant hearing screening programmes to complement those based in hospitals, especially in LMICs. Two significant studies in 2012 (Friderichs et al.) and 2016 (De Kock et al.) demonstrated the efficacy of maternity clinics for infant hearing screening in the Western Cape representing crucial advancements in early detection and intervention for congenital hearing loss.^31,36^ However, there has been a notable absence of systematic community-based infant hearing screening programmes within immunisation clinics across the Western Cape since then. This absence underscores a missed opportunity to continue the progress made in ensuring timely identification and support for infants at risk of hearing loss, highlighting the need for renewed efforts to prioritise such essential screening initiatives. Investigating the availability and accessibility of NHS services at PHC facilities in the CCT can provide invaluable insights into primary ear and hearing care service delivery. This could potentially contribute to future planning efforts aimed at improving primary ear and hearing care services in LMICs to mitigate the negative consequences associated with delayed diagnosis of hearing loss.

The findings from the study highlight a critical gap in the current NHS approach within South Africa, particularly at PHC facilities. The alarming statistic that none of the participants reported utilising the gold standard objective screening tests for hearing loss detection, such as OAEs or AABR, is deeply concerning. Moreover, the reliance on parental concerns or individual experience highlights a systemic issue leading to delayed detection and intervention for infants with hearing loss.^14^ The historical reliance on outdated methods, such as the rattle test, further compounds the problem, emphasising the urgent need for modern, standardised screening protocols.^37^ Similar findings regarding limited availability of NHS services were reported in studies conducted in other provinces of South Africa.^38,41,42^ Limited availability of NHS services at PHC facilities across South Africa is attributed to various factors such as lack of equipment and a shortage of trained personnel to conduct the screening.^41,42^ Because of limited availability of the NHS in South Africa, babies with hearing loss are often diagnosed very late. The potential for late diagnosis, as evidenced by the reported average age of diagnosis ranging from 23 to 44.5 months, emphasises the critical necessity for early detection to optimise intervention outcomes.^31^ A more recent study at a hospital in the Western Cape, South Africa, found that the mean age of diagnosis of permanent congenital or early-onset hearing loss was still only at 31.4 months.^39^ By this age, the critical period for intervention has nearly passed, rendering any subsequent efforts less effective in enhancing childhood communication development.^14,43^

Furthermore, the inconsistency in knowledge and the absence of standardised protocols for referral pathways highlight the urgent need for comprehensive training and guidelines. This lack of standardisation not only undermines the quality of care but also contributes to disparities in screening practices across different regions of South Africa. Similar findings were observed in a study by Kanji and Khoza-Shangase in 2019^40^ and Khan et al. in 2020,^44^ noting comparable inconsistencies in referral patterns for babies with suspected hearing loss between primary care clinics and higher levels of care in the NHS across various South African provinces. To enhance standardised hearing healthcare delivery across South Africa, well-designed care pathways are essential, allowing clinicians to focus on situational, interpersonal and intuitive aspects of medical care while minimising errors.^44^

Although most participants in this study expressed confidence in addressing primary ear and hearing care concerns, none had formal training on how to screen a baby’s hearing using objective screening tests, nor did they routinely offer preventive and promotional hearing health services. The lack of continued professional development for primary ear and hearing healthcare staff regarding the NHS is well-documented in previous studies.^45,46^ Participants’ acknowledgment of the importance of the NHS, coupled with their interest in acquiring additional training and knowledge, presents an opportunity for intervention. However, it is imperative to meet this interest with concrete actions, including implementing standardised training programmes and developing clear referral pathways.

In South Africa, healthcare workers at PHC clinics are likely the first point of contact to encounter a baby with suspected hearing loss or communication delay. Moving forward, efforts should focus on equipping healthcare workers at PHC clinics with the necessary tools and training to provide the NHS at routine immunisation clinics and improve hearing healthcare services for children. This includes adopting reliable, objective screening methods and the establishment of standardised testing protocols and referral pathways. Additionally, developing standard operating procedures and providing ongoing professional development opportunities are crucial for enhancing the quality and accessibility of NHS services across South Africa.

Addressing the limited availability and knowledge of NHS services highlighted in this study requires a concerted effort from healthcare authorities, policymakers and stakeholders. The findings highlight the importance of prioritising NHS services in PHC facilities and suggest standardising training on primary ear and hearing care for all healthcare workers, especially at mother and child healthcare clinics. Additionally, advocating for and raising awareness on hearing loss is recommended, along with developing guidelines for appropriate referrals and management of ear and hearing concerns. The implementation of these measures will ultimately improve primary ear and hearing care in infants and children, mitigating the negative consequences associated with late and unaddressed hearing loss. A limitation of this study was that participants did not have the option to complete the survey in their native language. Future research should offer participants the option to complete the survey in their native language, enhancing accessibility and ensuring more accurate data collection and representation. Future research should also focus on extending invitations to all healthcare workers within the facilities, thereby increasing the sample size. Another limitation of this study was relying solely on healthcare workers’ self-perceived awareness, knowledge and training in the field of primary ear and hearing care. Future research should evaluate healthcare workers’ actual training and skill set in these areas.

## Conclusion

The findings of this study revealed an absence of standardised NHS services across 26 PHC facilities within the CCT. The only hearing screening service provided at PHC facilities relied on verbal inquiries directed at parents and caregivers about their child’s hearing and communication development, utilising the Road to Health book developmental screening tool. There is also an absence of standardised referral pathways for infants presenting with hearing and communication delays. While the participants expressed a level of self-perceived confidence in addressing primary ear and hearing care concerns at PHC clinics, they lacked the knowledge, training and skill set on how to screen a baby’s hearing using objective testing methods. The findings of this study are pivotal, providing invaluable insights that should be integral to future planning efforts aimed at enhancing primary ear and hearing care services as well as improving the accessibility and availability of NHS services in PHC facilities across the Western Cape.
